# Citespace-based visualization of nutritional research hotspots for oncology patients

**DOI:** 10.3389/fnut.2026.1661764

**Published:** 2026-01-29

**Authors:** Na Zhang, Mei Li, Jing Zhao, Huijuan Wang, Jingna Wang, Honglin Niu

**Affiliations:** 1 First Department of Thoracic Radiotherapy, The Fourth Hospital of Hebei Medical University, Shijiazhuang, Hebei, China; 2 School of Nursing, Hebei Medical University, Shijiazhuang, Hebei, China; 3 Department of Abdominopelvic Radiotherapy, The Fourth Hospital of Hebei Medical University, Shijiazhuang, Hebei, China

**Keywords:** malignancy, malnutrition, nutritional support, visual analysis, bibliometrics

## Abstract

**Objective:**

To analyze the current status of nutrition-related research on cancer patients in the past 20 years, explore the current research hotspots and frontiers, and provide references for nutrition-related research on cancer patients.

**Methods:**

Literature on cancer nutrition published between 1 January 2004 and 31 July 2024 was retrieved from the Web of Science Core Collection and the China National Knowledge Infrastructure (CNKI). CiteSpace 6.4. R1 was used for bibliometric analysis.

**Results:**

From Web of Science, 954 English-language articles were included, yielding nine clusters: gastrointestinal neoplasms–enteral nutrition, nutritional screening and assessment (covering nutrition assessment, nutrition screening, and nutrition risk index), sarcopenia, nutritional prehabilitation, risk, advanced cancer, and patient-generated subjective global assessment. From CNKI, 1,156 Chinese-language articles were included, forming eight clusters: enteral/parenteral nutrition, relative angle, nurse-led interventions, nutritional support, prognostic nutritional index, patients with gastrointestinal tumors, enhanced recovery after surgery, and nutritional risk assessment. Research centered on gastrointestinal cancer and perioperative care, with hotspots in malnutrition screening and assessment, sarcopenia, enhanced recovery, and prognosis.

**Conclusion:**

Cancer nutrition research is progressing steadily, with a predominant focus on evaluating and screening patients’ nutritional status. Strengthening international and interdisciplinary collaboration and expanding the breadth and depth of inquiry will help advance this field.

## 1 Introduction

Cancer remains a major global public health challenge. WHO estimates for 2020 report 19.3 million new cases and almost 10 million deaths linked to cancer (1), and the worldwide burden is expected to rise for at least the next two decades. In China alone, 4.8247 million new cases and 2.5742 million cancer-related deaths were recorded in 2022 (2). Between 20 and 70% of patients develop malnutrition, nearly three-quarters are already malnourished at admission, and 55–85% of those with advanced disease face persistent malnutrition (3).

Malnutrition is a clinical state that compromises physical and psychological function and worsens outcomes (4). It undermines treatment efficacy, lengthens hospitalization, raises costs, and increases complication rates and mortality (5–7). These effects span the entire cancer trajectory, making nutritional assessment and timely intervention central to comprehensive care (8, 9). The field still needs broader and deeper inquiry to develop more effective nutrition strategies for patients with cancer (10).

CiteSpace enables bibliometric visualization through co-occurrence and clustering analyses to map hotspots and frontiers within defined time windows (11). We used version 6.4. R1 to profile literature on cancer nutrition. The Web of Science Core Collection was selected for its rigorously curated SCI/SSCI titles and complete citation metadata suitable for CiteSpace. To capture influential Chinese-language scholarship, we supplemented with CNKI, the most comprehensive source of core Chinese medical journals and theses. PubMed was not prioritized because it lacks the citation fields required for citation-network visualization, and Scopus offers limited coverage of Chinese oncology journals relative to CNKI. Combining WoSCC and CNKI allowed us to analyze global and domestic research within a unified framework.

## 2 Materials and methods

### 2.1 Literature search

We searched the Web of Science Core Collection for English-language literature and CNKI for Chinese-language literature. Advanced searches spanned 1 January 2004 through 31 July 2024; all retrievals were finalized on 2 August 2024, and no later publications were added.

For WoSCC, the query was (TS = cancer OR tumors) AND (TS = nutrition OR nutritional status) AND (TS = nutritional assessment) AND (TS = malnutrition). Records were limited to Science Citation Index Expanded and Social Sciences Citation Index, document types “Article” or “Review,” and English language, yielding 2,996 papers.

For CNKI, the subject query was (tumor + cancer patient + malignant tumor) AND (nutrition + malnutrition + nutritional status + nutritional support + nutritional management + nutritional assessment). We included only core academic journals (Peking University Core, CSSCI, CSCD) in Chinese, retrieving 1,334 papers.

### 2.2 Inclusion and exclusion criteria

Inclusion Criteria: (1) Papers related to the nutrition of cancer patients; (2) Published papers with full-text availability.

Exclusion Criteria: (1) Papers with missing information or duplicate papers; (2) Online-published papers, conference papers, books, and news; (3) Irrelevant papers.

To keep WoSCC and CNKI outputs comparable, we analyzed only peer-reviewed articles and reviews. Conference proceedings were excluded because they rarely provide standardized affiliations, structured keywords, or citation data in either database, which weakens co-word clustering and citation-burst detection. Proceedings also duplicate findings that later appear as journal articles, inflating counts of emerging topics. For CNKI, we further limited retrieval to Peking University core journals, CSSCI, and CSCD titles, which applies consistent editorial standards, supplies complete metadata (funding, affiliations, author information), and avoids institutional bulletins that lack external peer review. These filters produced a dataset with parallel rigor across both language sources.

After independent screening by two researchers, eligible papers were imported into CiteSpace 6.2. R4. The analysis covered January 2004 through July 2024 with one-year time slices. The g-index k-value was 25, Top N was 50, and pruning applied Pathfinder, pruned sliced networks, and pruned merged networks, with all other parameters left at defaults. Nodes analyzed included authors, countries, institutions, and keywords.

## 3 Results

### 3.1 Analysis of annual publication volume

**Figure 1:**
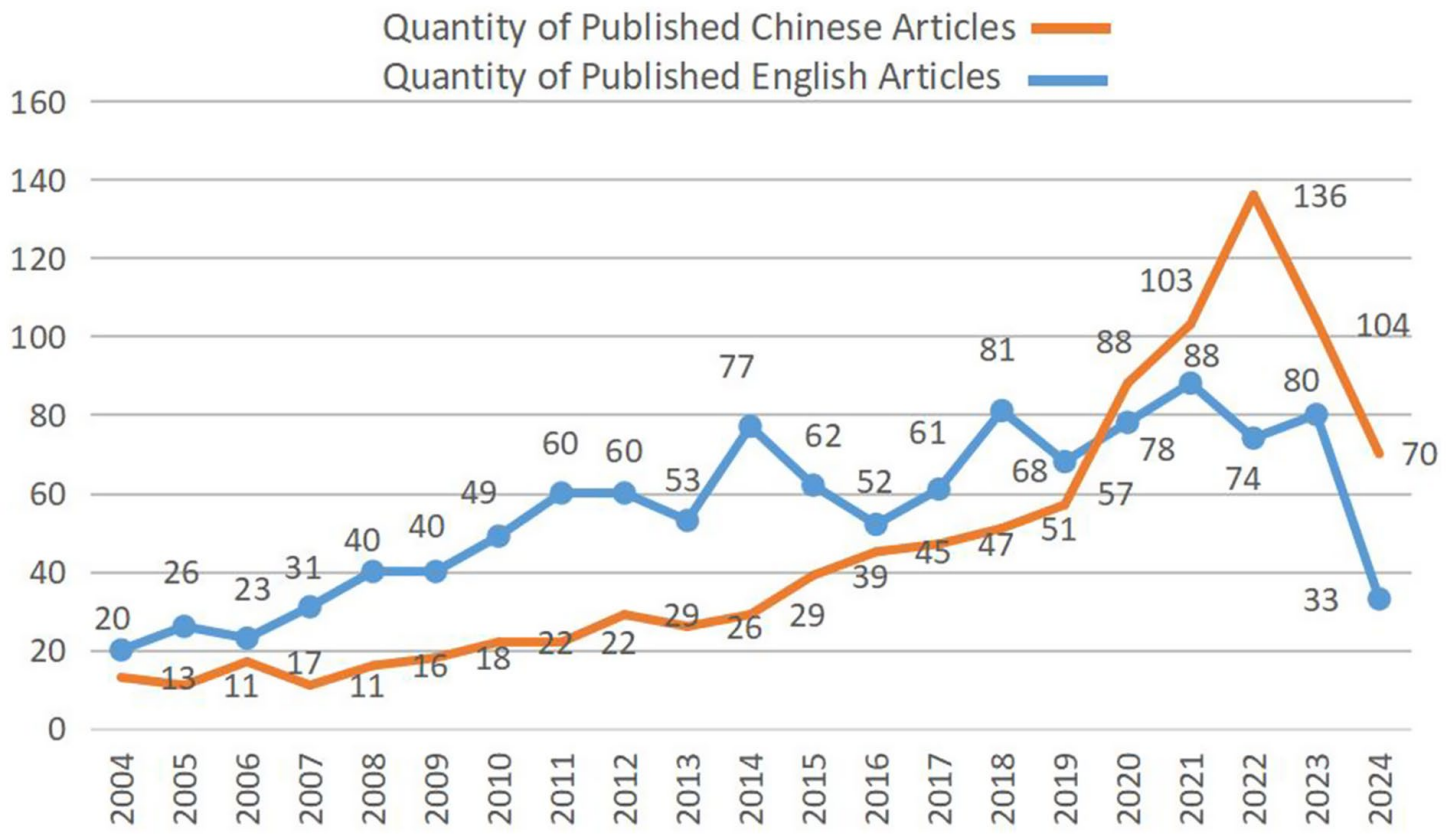
Publication trends in the field of nutrition for cancer patients from 2004 to 2024.

We ultimately included 954 English-language and 1,156 Chinese-language papers. The five leading publishing countries were China (193), the United States (155), Australia (94), Italy (76), and Brazil and Canada (66 each). Publications rose steadily, with English-language output peaking at 136 papers in 2022 before easing to 104 in 2023; volumes from 2021 through 2023 plateaued at this higher level (Figure 1).

### 3.2 Co-occurrence network analysis of research authors

**Figure 2:**
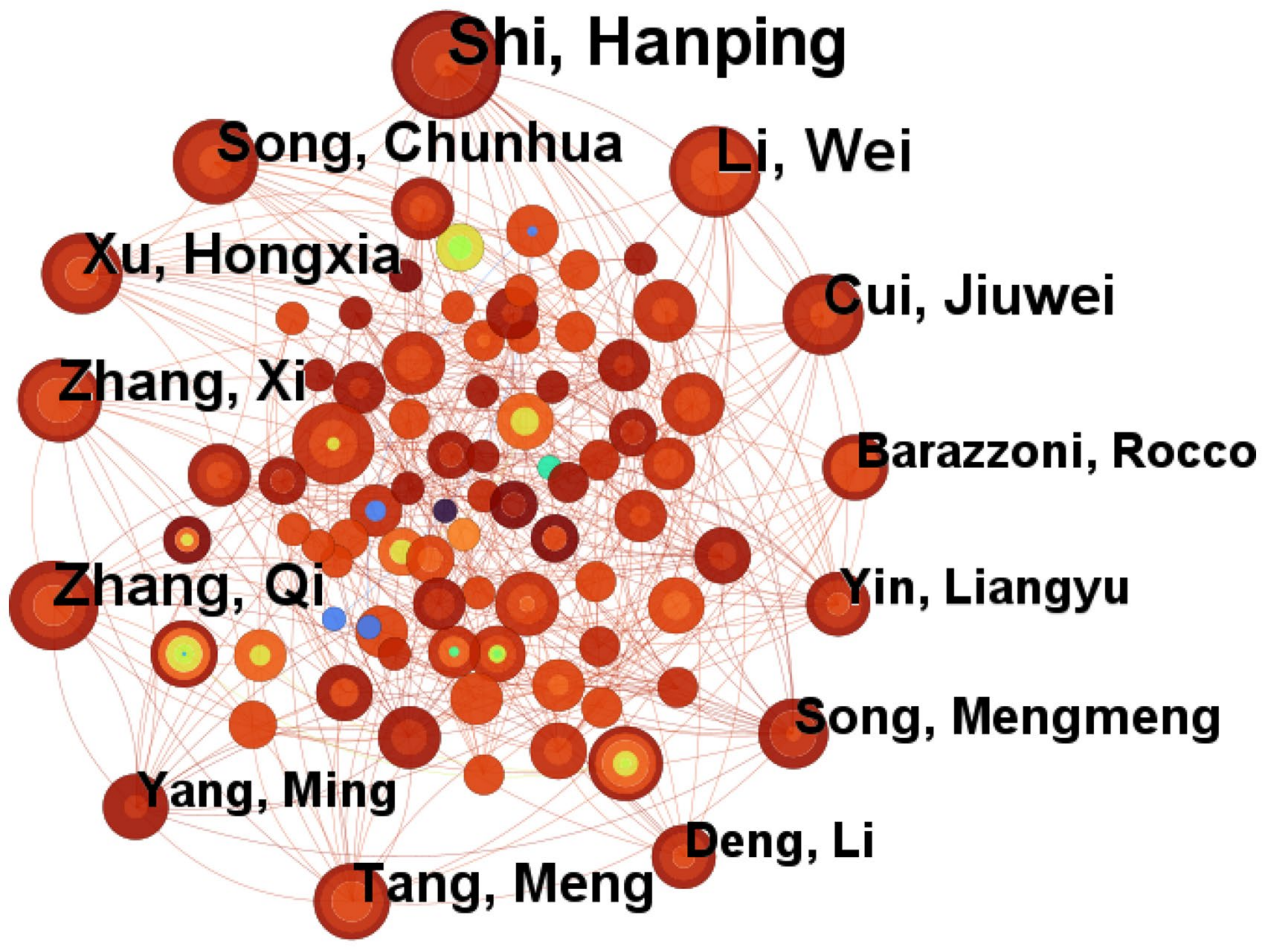
Co-author network map of English-language cancer nutrition research from 2004 to 2024.

**Figure 3:**
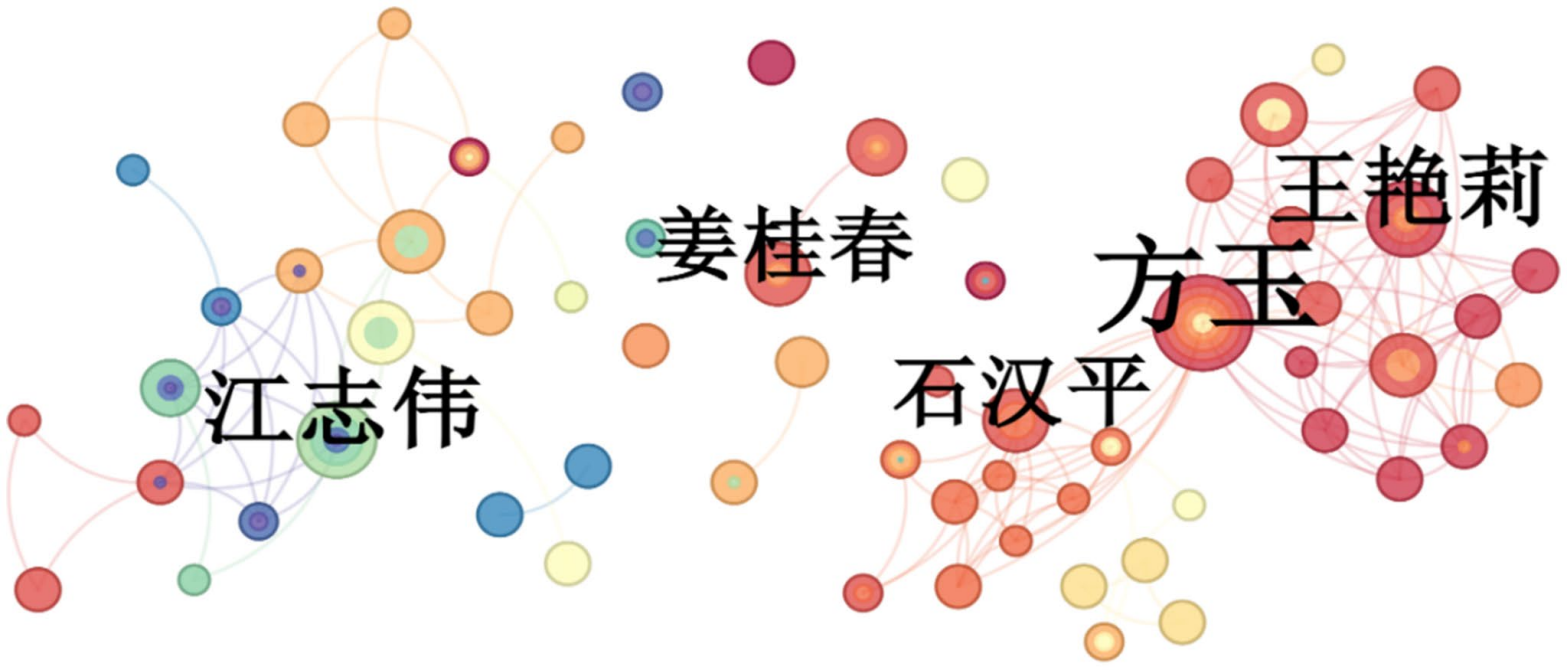
Co-author network map of non-English-language cancer nutrition research from 2004 to 2024.

We mapped co-authorship to identify influential researchers and collaboration structures. The English-language dataset produced a network of 606 authors connected by 1,100 links (Figure 2), reflecting dense collaboration. The Chinese-language dataset generated 718 authors and 805 links (Figure 3), indicating more fragmented partnerships. Node size corresponds to coauthor influence within the tumor-nutrition domain.

Applying Price’s Law [*N* = 0.749√n_max, where n_max is the output of the most prolific author (12)] yielded a threshold of four publications for the English-language set. Sixty-three core authors met this bar and produced 421 papers; Shi Hanping led with 22 publications, followed by Zhang Qi and Li Wei with 15 each (Figure 2). For CNKI papers, the threshold was three publications, identifying 38 core authors with 140 papers in total; Fang Yu authored 10, while Wang Yanli and Jiang Zhiwei published six apiece (Figure 3). China’s position as the most prolific country in WoSCC underscores its central role in advancing tumor-nutrition research.

### 3.3 Institutional co-occurrence network analysis

**Figure 4:**
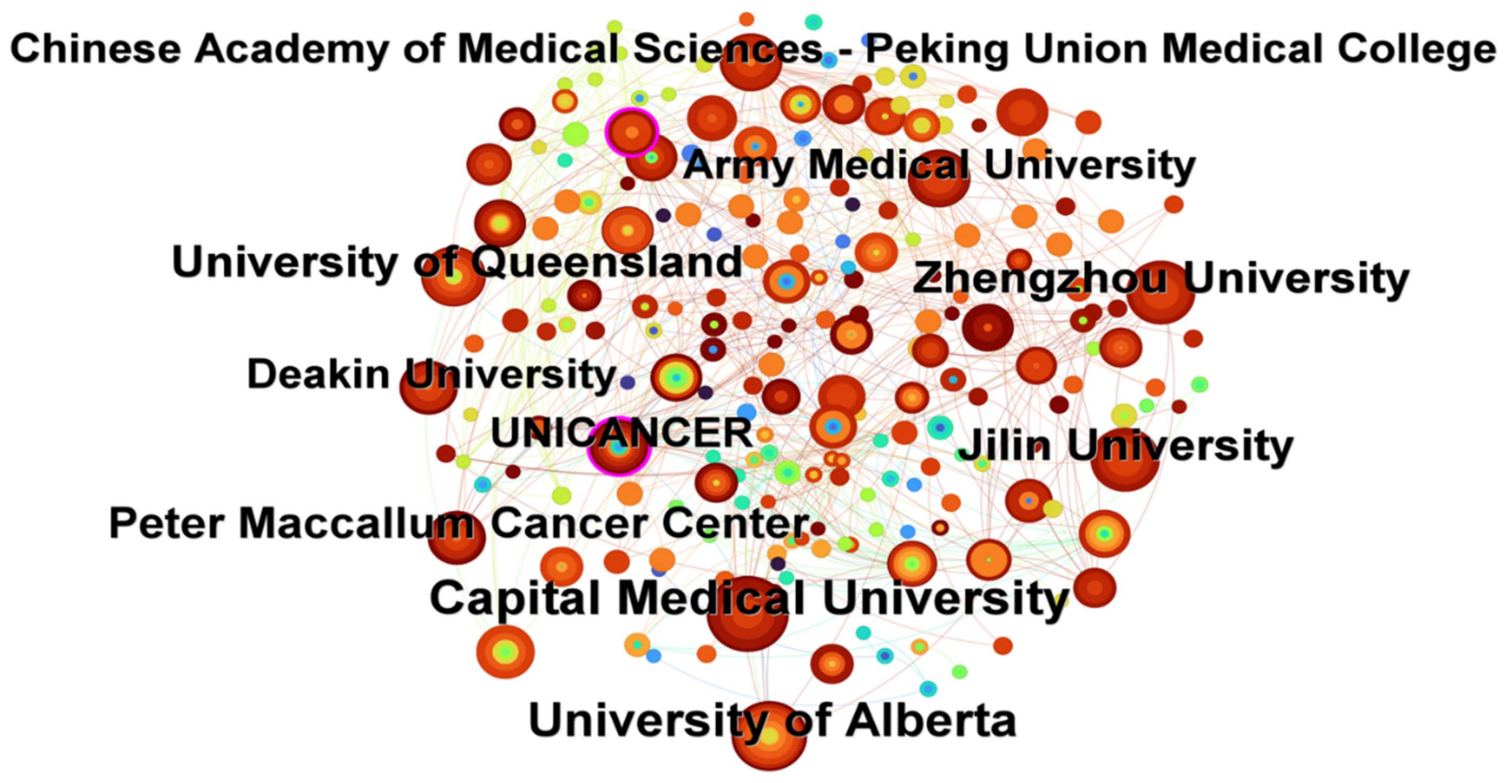
Institutional network map of English-language cancer nutrition research from 2004 to 2024.

Country-level collaboration shows a clear geographic concentration: structured cancer nutrition programs remain largely anchored in Europe. In the English-language sample, the most productive institutions were Capital Medical University, China (32 publications); University of Alberta, Canada (32); University of Queensland, Australia (24); Zhengzhou University, China (24); and Peter MacCallum Cancer Centre, Australia (21). More than 80% of the top 10 institutions were based in Western countries, yet the two highest-output organizations were Chinese and American (Figure 4 and Table 1). In the Chinese-language dataset, leading contributors were Xiangya Hospital of Central South University (16 publications), the Institute of General Surgery at Nanjing General Hospital of Nanjing Military Command (12), Ruijin Hospital affiliated with Shanghai Jiao Tong University School of Medicine (10), Shanxi Medical University (8), and Peking University Cancer Hospital (8) (Table 2).

**Table 1:** Top 10 institutions ranked by the number of publications in English-language cancer patient nutrition research from 2004 to 2024.

Serial number	Frequency	Centrality	Year	Institution
1	32	0.07	2021	Capital Med University
2	32	0.13	2006	University of Alberta
3	24	0.01	2013	University of Queensland
4	24	0.01	2020	Zhengzhou University
5	21	0.01	2016	Peter MacCallum Cancer Centre
6	19	0.01	2010	Deakin University
7	18	0.01	2014	Bond University
8	14	0.01	2013	Princess Alexandra Hospital
9	13	0.01	2021	First Hospital Jilin University
10	13	0.02	2015	Fondazione IRCCS Policlin San Matteo

**Table 2:** Top 10 institutions ranked by the number of publications in non-English-language cancer patient nutrition research from 2004 to 2024.

Serial number	Frequency	Year	Institution
1	16	2005	Xiangya Hospital of Central South University
2	12	2008	Institute of General Surgery of the People’s Liberation Army, Nanjing General Hospital of Nanjing Military Region
3	10	2007	Ruijin Hospital Affiliated to Shanghai Jiao Tong University School of Medicine
4	8	2020	Shanxi Medical University
5	8	2018	Cancer Hospital of China Medical University
6	7	2011	Peking University Cancer Hospital
7	7	2014	School of Nursing, Shanghai Jiao Tong University
8	7	2010	The Affiliated Gulou Hospital of Nanjing University
9	6	2009	Cancer Hospital, Chinese Academy of Medical Sciences
10	6	2012	Peking Union Medical College Hospital, Chinese Academy of Medical Sciences

### 3.4 Keyword analysis

#### 3.4.1 Co-occurrence network analysis of keywords

**Figure 5:**
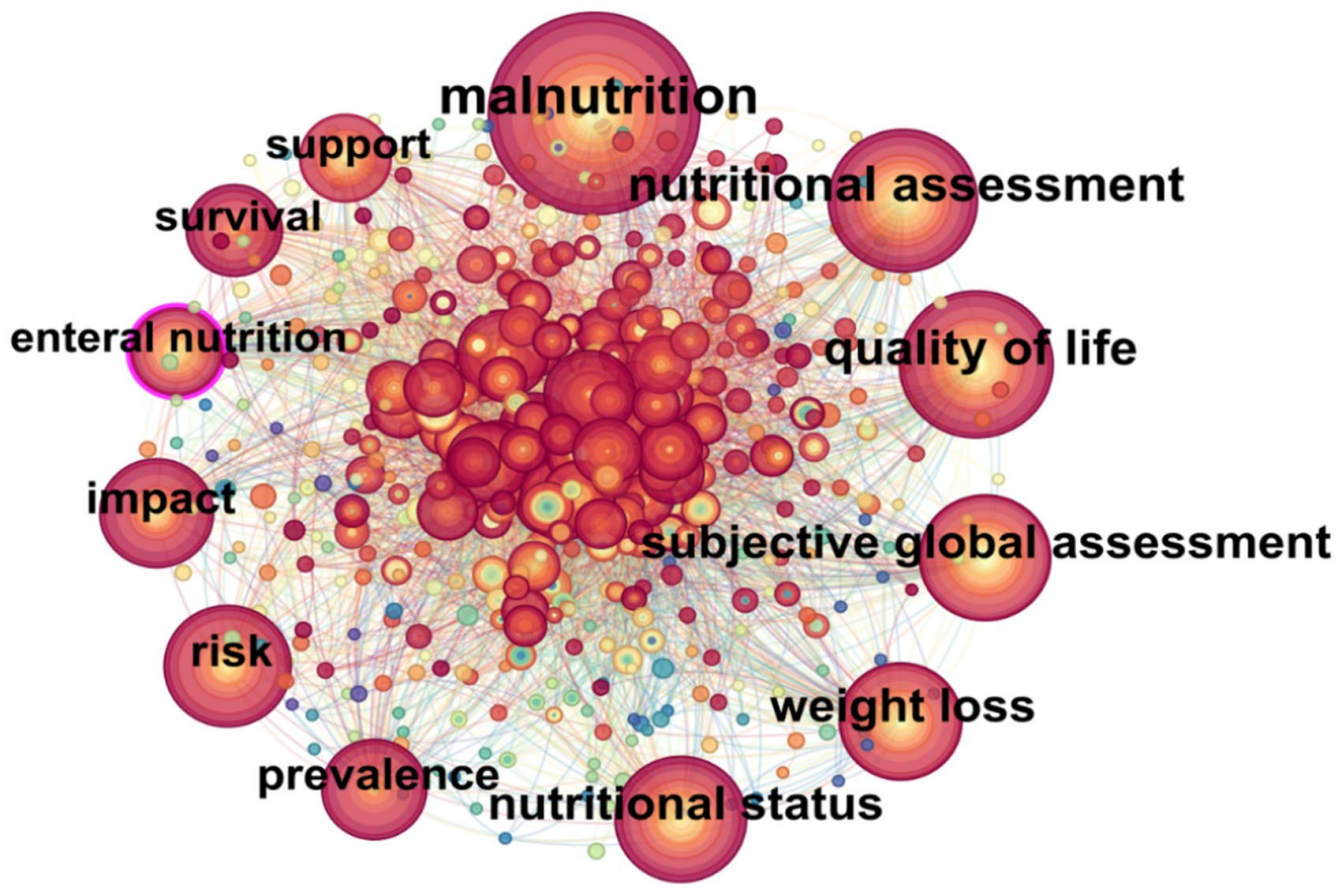
Co-occurrence network map of keywords in English-language cancer nutrition research from 2004 to 2024.

**Figure 6:**
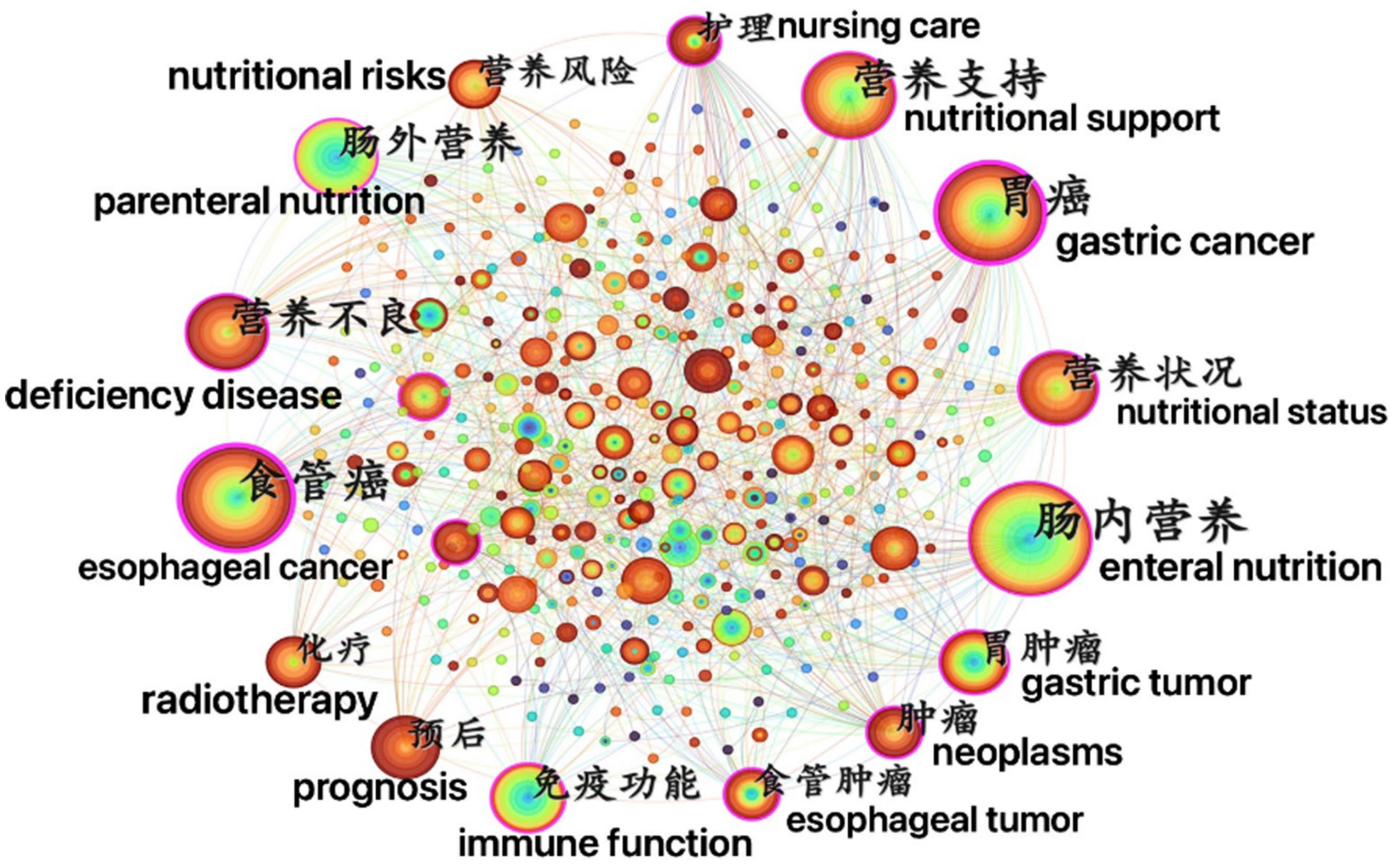
Co-occurrence network map of keywords in non-English-language cancer nutrition research from 2004 to 2024.

Keywords are a high-level summary of the research content of an article. They can intuitively reveal the high-frequency and high-centrality research keywords in studies related to tumor nutrition which helps to analyze the research hotspots in this field. Keyword preprocessing combined synonyms (“nutrition assessment” and “nutritional assessment”) so that identical concepts were counted once. The co-occurrence maps of keywords related to tumor nutrition show that for English-language papers, 496 keyword nodes and 1,011 connections were generated, while for non-English-language papers, 522 keyword nodes and 2,167 connections were generated (Figures 5, 6).

According to the formula for defining high- and low-frequency words proposed by Donohue (1973) (13):

$T=\frac{\sqrt{1+8I}-1}{2}$ (where *I* is the frequency of the most frequently occurring keyword).

Keywords with an occurrence frequency greater than or equal to *T* are defined as high-frequency words. The calculated minimum occurrence frequencies for high-frequency words are 20 times for non-English papers and 28 times for English papers. The top 20 high-frequency keywords are presented in Table 3.

**Table 3:** High-frequency keywords (top 20) in cancer patient nutrition research from 2004 to 2024.

Serial number	Non-English keywords	Frequency	Centrality	English keywords	Frequency	Centrality
1	Enteral nutrition	205	0.24	Malnutrition	382	0.04
2	Gastric cancer	157	0.23	Quality of life	206	0.06
3	Esophageal cancer	143	0.21	Subjective global assessment	188	0.05
4	Nutritional support	118	0.22	Weight loss	174	0.08
5	Malnutrition	92	0.16	Nutritional status	164	0.05
6	Nutritional status	84	0.12	Prevalence	147	0.04
7	Parenteral nutrition	84	0.09	Risk	140	0.01
8	Tumor	67	0.14	Impact	123	0.03
9	Gastric tumor	62	0.08	Nutrition assessment	116	0.03
10	Immune function	60	0.06	Enteral nutrition	112	0.1
11	Prognosis	56	0.07	Survival	106	0.03
12	Chemotherapy	50	0.06	Nutritional assessment	94	0.04
13	Nutritional risk	48	0.11	Support	91	0.06
14	Esophageal tumor	47	0.11	Chemotherapy	89	0.08
15	Nursing care	43	0.05	Mortality	88	0.06
16	Nutrition	39	0.1	Outcome	76	0.02
17	Perioperative period	38	0.02	Cancer	75	0.05
18	Prognostic nutritional index	38	0.05	Body composition	72	0.08
19	Quality of life	35	0.03	Cachexia	70	0.02
20	Complications	35	0.03	Nutrition	67	0.02

Betweenness centrality represents the number of times a node in the network serves as a bridge on the shortest path between two other nodes. Nodes with high betweenness centrality may be located between two large clusters or sub-networks and are shown with a purple ring. The thicker the purple ring, the stronger the centrality. The centrality of a node indicates its importance. A value exceeding 0.1 suggests that the node is a key node, and the larger the value, the greater its importance and influence, with closer connections between nodes (14).

In English-language papers, the keywords “malnutrition,” “quality of life,” and “subjective global assessment” appear most frequently. In non-English research, the top three keywords in terms of centrality strength in this field are “enteral nutrition” (0.24), “gastric cancer” (0.23), and “esophageal cancer” (0.21). Therefore, we can preliminarily conclude that enteral nutrition for malnourished patients with esophageal and gastric cancer is a key area of concern.

#### 3.4.2 Keyword cluster analysis

Keyword cluster analysis reflects the collection of research topics in a specific field during a particular period. The cluster number is inversely proportional to the cluster size, meaning that the smaller the number, the larger the cluster.

Visual inspection of the CiteSpace cluster tree showed that the subclusters originally labeled ‘nutrition assessment,’ ‘nutrition screening,’ and ‘nutrition risk index’ shared the same core nodes (PG-SGA, NRS-2002, GLIM, nutrition risk score) and thus represented a single research theme. To avoid redundancy, we manually merged them into one cluster, now termed ‘nutritional screening and assessment,’ which integrates the various screening workflows, assessment tools, and risk indices. The final cluster list therefore reads: ‘gastrointestinal neoplasms–enteral nutrition,’ ‘nutritional screening and assessment,’ ‘sarcopenia,’ ‘nutritional prehabilitation,’ ‘risk,’ ‘advanced cancer,’ and ‘patient-generated subjective global assessment,’ maintaining nine clusters in total.

**Figure 7:**
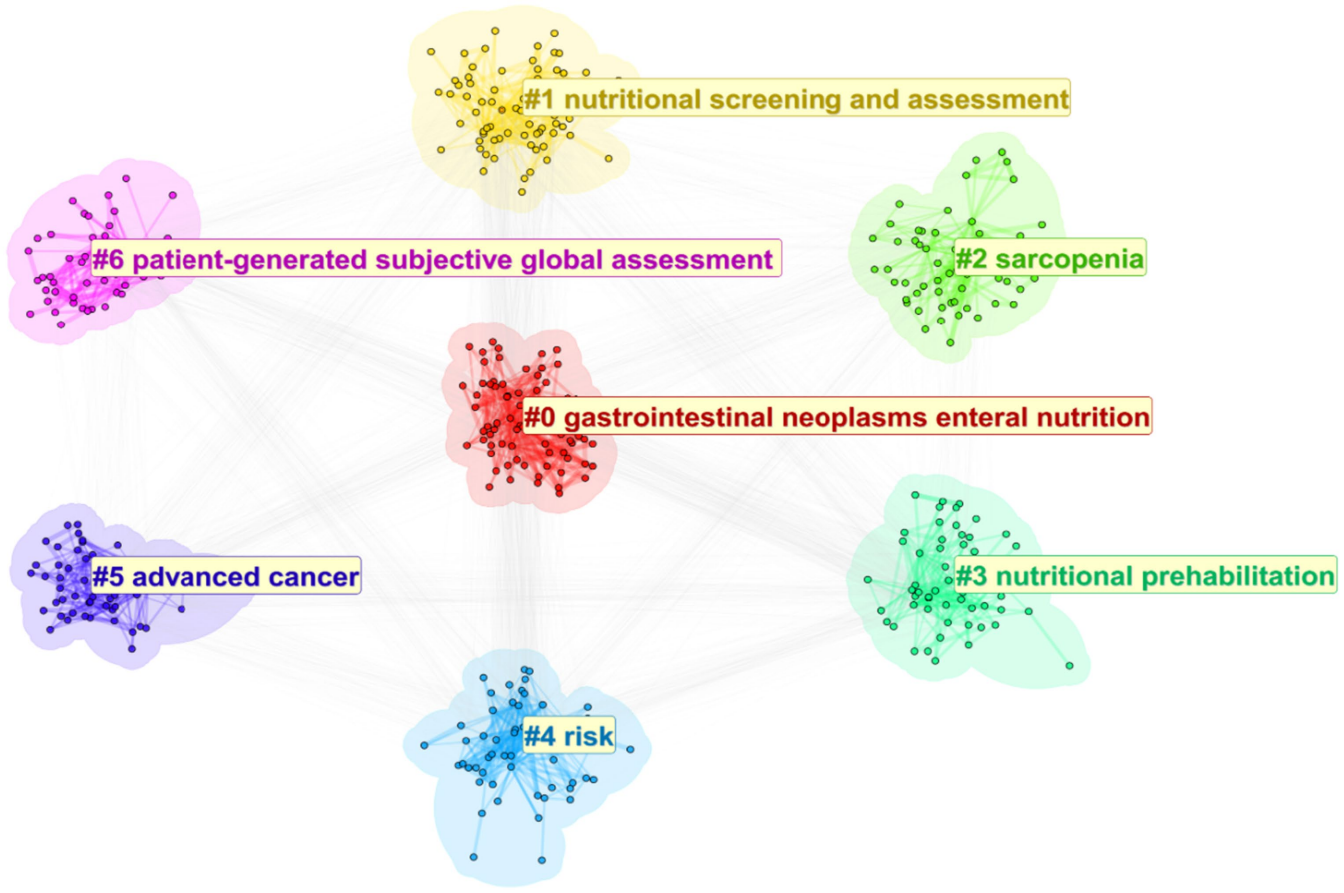
Keyword clustering network map of English-language cancer nutrition research from 2004 to 2024.

The modularity value (Modularity Q) of the English cluster map is 0.2894, and the weighted mean silhouette value (S) is 0.6379. These values indicate that the clustering results are reliable (15) (Figure 7).

**Figure 8:**
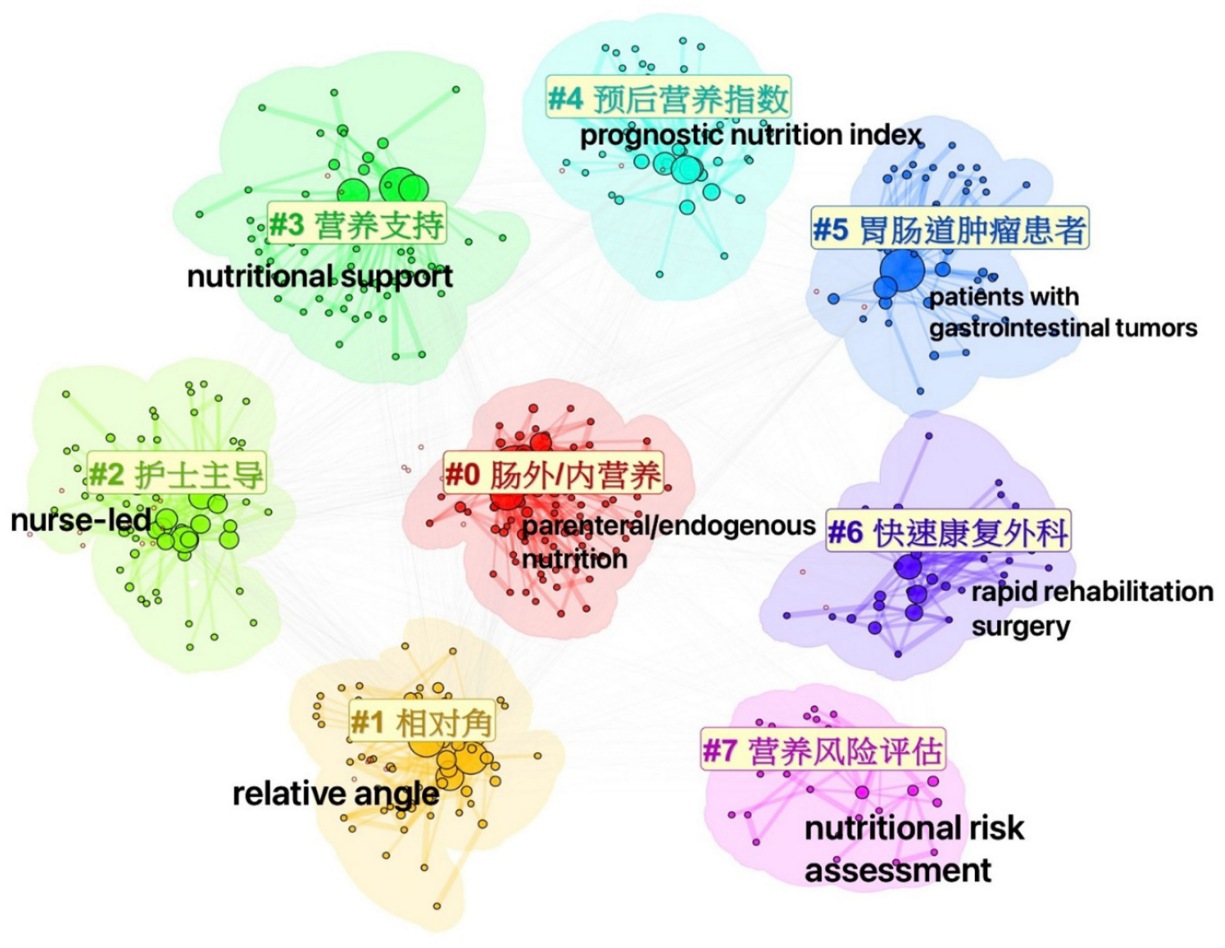
Keyword clustering network map of non–English–language cancer nutrition research from 2004 to 2024.

Non-English keyword clustering yielded eight themes—enteral/parenteral nutrition, relative angles, nurse-led initiatives, nutritional support, prognostic nutritional index, gastrointestinal-tumor patients, enhanced recovery after surgery, and nutritional risk assessment. The map’s Modularity *Q* = 0.3957 and silhouette *S* = 0.7648 confirm well-separated clusters (Figure 8), yet their limited cross-links highlight how Chinese tumor-nutrition research remains fairly siloed, leaving considerable room for broader integration.

#### 3.4.3 Keyword burst analysis

**Figure 9:**
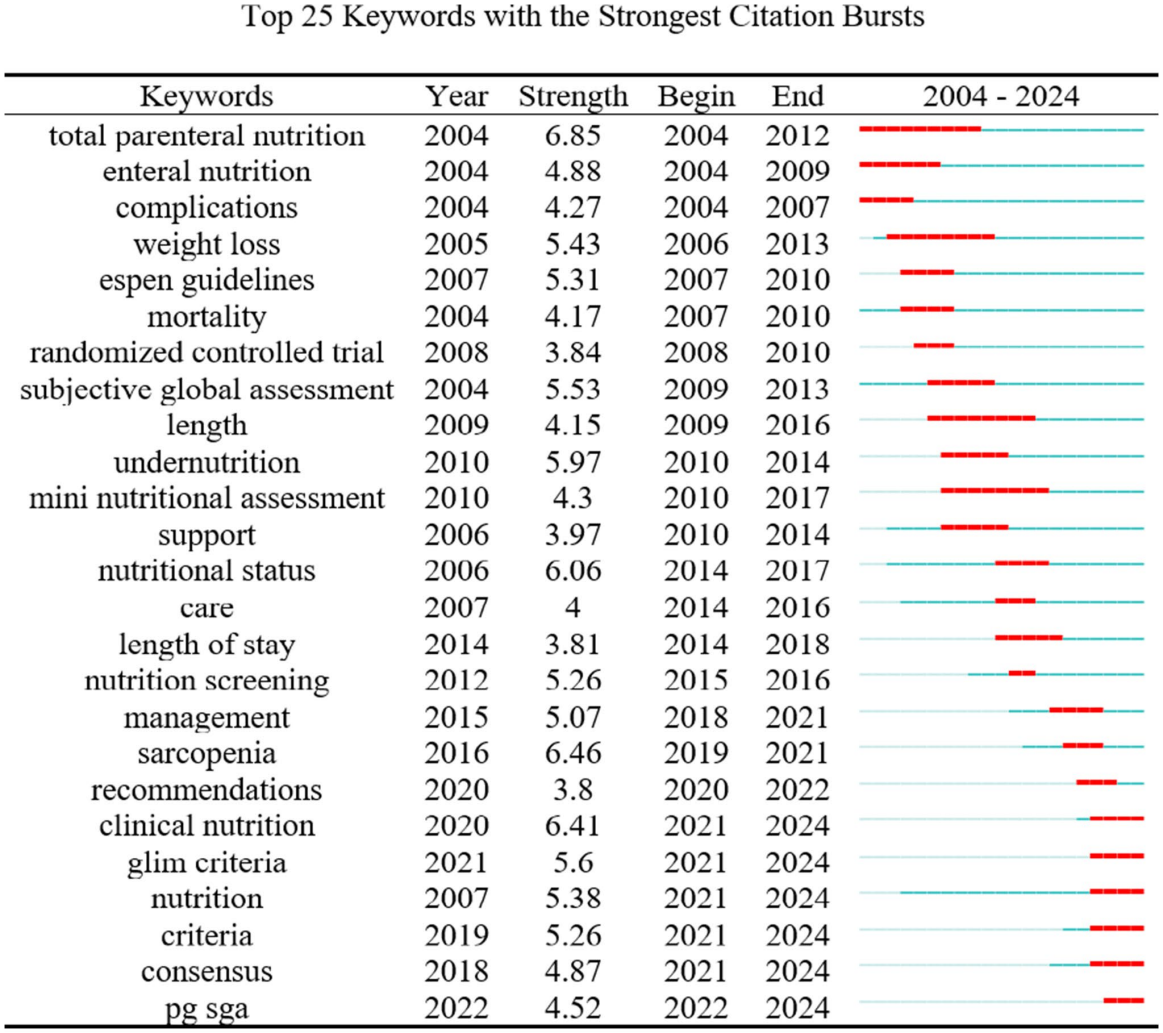
Top 25 emerging keywords in cancer patient nutrition research in English-language literature from 2004 to 2024.

**Figure 10:**
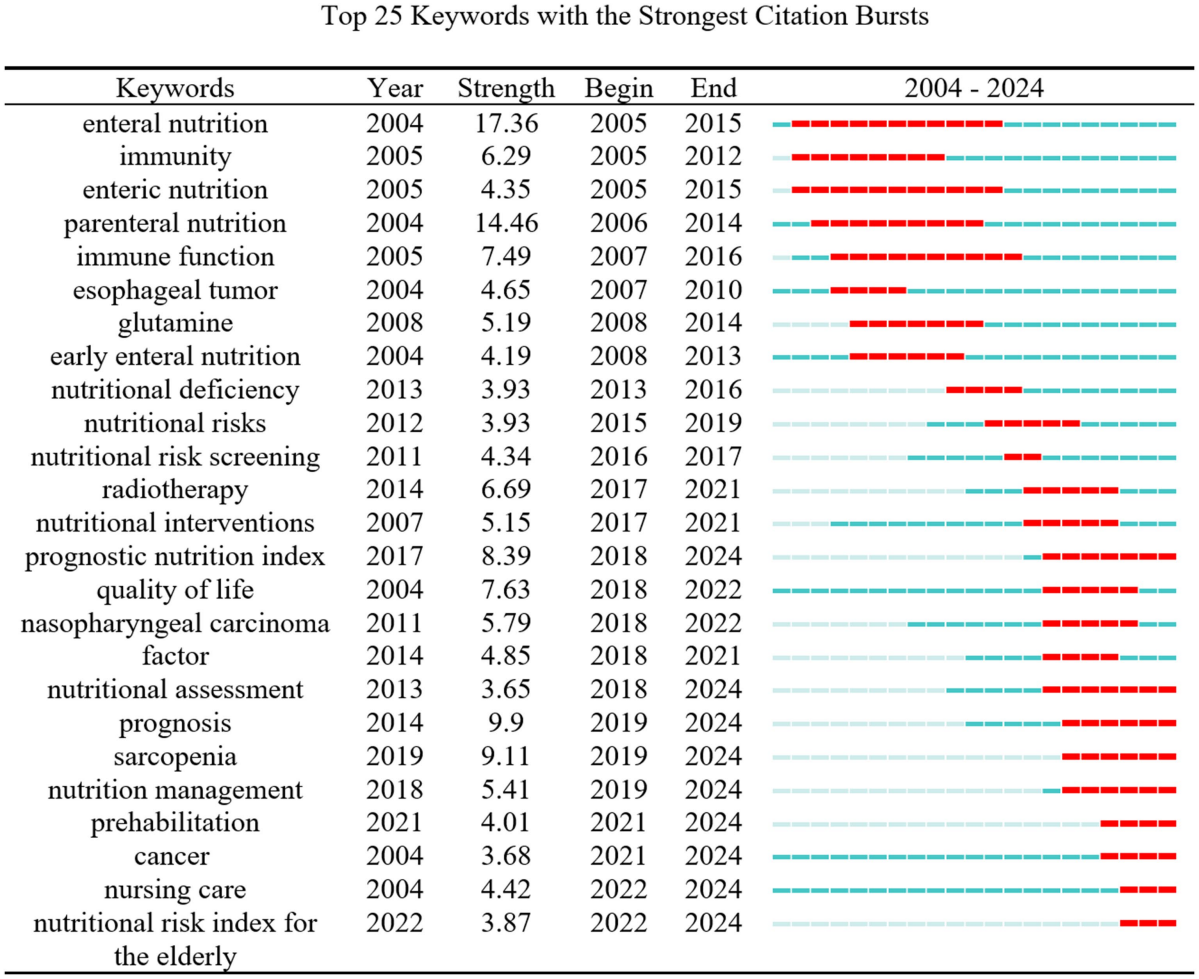
Top 25 emerging keywords in cancer patient nutrition research in non-English-language literature from 2004 to 2024.

Keyword burst detection (minimum duration = 2, top 25 co-cited terms over 20 years) tracks shifting hotspots. In English-language studies, “clinical nutrition” and “GLIM criteria” lead the frontier; “clinical nutrition” has shown a burst strength of 6.41 over the past 3 years and remains a fast-moving topic (Figure 9). In the Chinese-language set, “prehabilitation,” “tumor nursing,” and “geriatric nutritional risk index” headline current research, while “sarcopenia” registered a five-year burst strength of 9.11, signaling sustained momentum in tumor-nutrition studies (Figure 10).

### 3.5 Journals and authoritative literature

Across the 1,278 English-language records retrieved from WoSCC, 162 journals published work on oncology nutrition. Output was concentrated in a small cohort of titles: Supportive Care in Cancer (124 papers, 9.7% of total) and Clinical Nutrition (108 papers, 8.5%) led publication volume, followed by Nutrition and Cancer (96 papers), European Journal of Clinical Nutrition (72 papers), and Nutrition (61 papers). These journals also showed above-average citation impact (mean citations per article ≥24), underscoring their role as core outlets for disseminating nutritional screening tools, body-composition monitoring, and perioperative nutrition strategies.

Highly cited articles within this corpus mainly addressed standardized assessment frameworks and prognostic nutrition indices. The GLIM international consensus update (Cederholm et al. (36), Clinical Nutrition; 1,268 citations) established a two-step approach that couples screening with phenotypic/etiologic criteria, forming the methodological backbone for many recent studies. Prado et al. (37, Lancet Oncol.; 1,071 citations) demonstrated that sarcopenic obesity markedly increases chemotherapy toxicity and overall mortality, prompting routine CT-based body-composition monitoring. Arends et al. (38, ESPEN Guideline; 963 citations) synthesized evidence on metabolic derangements, screening intervals, and enteral/parenteral support thresholds for cancer patients. Additional influential works include Wongdama et al. (39, Medicine (Baltimore); 612 citations) validating PG-SGA short-form cutoffs for radiotherapy candidates, and Furuke et al. (40, Cancer Diagn Progn; 548 citations) proposing nutrition risk index thresholds that predict survival in gastric cancer. Collectively, these high-impact contributions clarify why nutritional screening and assessment remain central clusters in our network analysis: they provide the standardized instruments and clinical endpoints that link malnutrition, treatment tolerance, and long-term prognosis.

## 4 Discussion

### 4.1 Analysis of the current status of tumor nutrition research

This review confines the observation window to the most recent two decades for two pragmatic reasons. First, the early 2000s mark the global dissemination of PG-SGA, NRS-2002, early ESPEN guidance, and subsequent GLIM criteria, which triggered a steep rise in oncology-nutrition publications that encapsulate current clinical philosophy. Second, Web of Science records before 2004 are sparse and often lack standardized keywords, which would dilute cluster interpretability and compromise co-word accuracy. Keeping the focus on 2004–2024 therefore balances data completeness with analytic clarity, allowing the network to mirror contemporary clinical practice and research momentum.

Prior bibliometric snapshots of oncology nutrition have largely targeted narrow subdomains—such as parenteral nutrition, perioperative support for single tumor types, or cachexia-specific cohorts—and most were limited to short windows with descriptive indicators only. As a result, they could not place nutritional screening instruments, body-composition monitoring, and peri-treatment support strategies within one longitudinal network. By assembling 1,278 WoSCC records published between 2004 and 2024 and applying CiteSpace timeline views, betweenness-centrality mapping, and burst detection, our study provides the first integrated map that links guideline milestones (PG-SGA, NRS-2002, ESPEN, GLIM) to emerging clusters such as sarcopenia and nutritional prehabilitation. This consolidated approach clarifies how research priorities have shifted from simple malnutrition screening toward multidimensional risk stratification and metabolic management, thereby filling the gap left by earlier, more fragmented bibliometric analyses.

In this study, through bibliometric analysis, it can be seen that in recent years, the number of publications related to the nutrition of tumor patients has generally shown an increasing trend. The number of English-language publications reached its peak in 2022 (136 articles). The number of non-English publications reached its peak from 2021 to 2023 and then entered a stable state, indicating that the attention in this field continues to grow.

Countries such as China and the United States have conducted extensive research in this field and have high influence, with their publication volumes far exceeding those of other countries. Firstly, the high number of publications from China may be due to the relatively high incidence and mortality rates of esophageal and gastric cancers in China. Approximately 47.42% of esophageal and gastric cancers occur in China, and the 5-year survival rate of patients remains at around 30% (16). On the other hand, it may be because this study only retrieved the Web of Science database, resulting in a relatively small number of literatures.

Although China (412 papers) and the United States (376 papers) dominate publication volume, the co-authorship network reveals a hub-and-spoke pattern rather than a tightly knit global web. Most high-output Chinese institutions collaborate domestically, and only a handful of cross-border ties link them to U. S. comprehensive cancer centers. This asymmetry suggests that national funding streams—China’s Key R&D Program and the U. S. NIH/NCI supportive-care portfolio—prioritize internal capacity building over shared multicenter trials. It also explains why Western Europe, despite strong ESPEN leadership, contributes fewer high-betweenness nodes: funding is fragmented across smaller consortia, which limits the visibility of their outputs in WoSCC. Encouraging joint calls or shared registries could therefore accelerate the translation of nutritional screening tools into globally comparable outcome studies.

In terms of publication institutions and regions, Western developed countries such as the United States, the United Kingdom, and Canada have extensive cooperation. Capital Medical University and Zhengzhou University in China rank among the top in terms of publication volume, but they have relatively few cooperative relationships with other institutions. Currently, a relatively stable cooperation network has been formed among authors and institutions. In the future, interdisciplinary, inter-institutional, cross-regional, and international team collaborations should be further strengthened to improve the quality of scientific research results in tumor-related nutrition research.

### 4.2 Analysis of research hotspots and frontiers in tumor nutrition

Currently, the high-frequency keywords in research related to the nutrition of tumor patients mainly focus on patients with gastrointestinal tumors and those in the peri-operative period (17–19). Keyword burst and cluster analyses show that the research hotspots include the assessment and management of nutritional status, sarcopenia, prehabilitation, and prognosis.

Linking the four largest keyword clusters to clinical action clarifies why they persist as hotspots. Malnutrition and sarcopenia clusters highlight the ongoing gap between guideline-endorsed screening (PG-SGA, GLIM) and real-world uptake: fewer than 40% of trials report routine body-composition assessments, underscoring the need for hospital policies that tie reimbursement or quality metrics to validated screening tools. The perioperative nutrition cluster shows that Enhanced Recovery protocols now integrate immunonutrition and carbohydrate loading, yet adherence remains uneven because insurance schemes often categorize these products as optional supplements. Prehabilitation-related keywords cluster around exercise-nutrition bundles before chemotherapy or radiotherapy, suggesting that preventive funding streams—rather than inpatient budgets—must be engaged to scale such programs. Together, these clusters argue for policy levers that convert bibliometric hotspots into standardized care pathways, aligning trial design, reimbursement, and quality indicators.

Burst detection around ‘sarcopenia’ beginning in 2018 coincides with two practice shifts: GLIM formally reclassified low muscle mass as a diagnostic criterion for malnutrition, and automated CT-based muscle quantification moved from research to routine staging in gastrointestinal and hepatobiliary tumors. These changes made sarcopenia measurable at scale, so trials increasingly stratify patients by skeletal muscle index when tailoring perioperative feeding or chemotherapy dosing. The recent burst of ‘clinical nutrition’ reflects reimbursement reforms (e.g., China’s DRG pilots, EU malnutrition screening mandates) that push hospitals to integrate dietitians into oncology teams and to document nutrition-specific outcomes. Consequently, research funding now favors trials that link nutritional interventions to survival or cost metrics rather than surrogate biochemical markers. The burst terms therefore trace how policy and technology upgrades redirected research priorities from exploratory nutrient studies to implementation-oriented clinical pathways.

The assessment and intervention of the nutritional status of tumor patients are one of the core issues in current research. Malnutrition ranks highest after the keywords “nutrition” and “cancer.” In treatment, malnutrition screening is of crucial importance. Nurses play an important role in nutrition screening and assessment (20, 21). More accurate and rapid assessment tools should be developed to identify and intervene in malnutrition at an early stage, so as to achieve personalized nursing in clinical practice.

Reber et al. (9) retrospectively analyzed patients with locally advanced esophageal and lung cancers who received concurrent chemoradiotherapy and found that weight loss was common. Malnutrition has been proven to be associated with poor outcomes. Li et al. (22) pointed out in their study that nutritional intervention can reduce the side effects (vomiting, mucositis) of neoadjuvant chemoradiotherapy, improve the nutritional status of patients, and shorten the postoperative hospital stay. Nutritional risk screening helps to identify and intervene in patients at high nutritional risk at an early stage. Continuous nutritional management may be the key to controlling cancer.

Scientific and reasonable diets and immunonutrition supplementation (such as *ω*-3 fatty acids, glutamine, trace elements, probiotics, etc.) can regulate the immune function of tumor patients (23), change the tumor microenvironment, improve body weight and the chronic inflammatory state of the body, reduce cancer risk, and improve the prognosis of tumor patients (24, 25). In recent years, the combination of traditional Chinese medicine and modern nutritional therapy has received extensive attention. It has unique advantages in regulating the overall immune function of tumor patients, improving their physical condition, and reducing the side effects of treatment (26).

With the increasing attention to tumor prevention and treatment, the research environment and conditions are gradually improving. Prehabilitation has been proven to have good effects in peri-operative patients, effectively reducing the occurrence of complications, improving the quality of life, and improving the long-term prognosis of patients, which is worthy of wide promotion (27).

Research on tumor-related sarcopenia has also gradually attracted attention (28). There is a strong connection between muscle mass and malnutrition, especially in patients with early-stage gastrointestinal tumors. The level of malnutrition, low BMI, and high nutritional risk significantly predict muscle mass loss (29). As malnutrition and the disease stage worsen, the patient’s tolerance decreases, which is closely related to poor treatment outcomes and prognosis (30). Therefore, in clinical practice, the assessment and screening of sarcopenia in tumor patients should be strengthened to improve the treatment effect.

It is estimated that by 2050, the number of cancer patients aged 80 and above will double (31). Elderly patients are more likely to be affected by age-related physiological changes and cancer-inducing factors and have a higher risk of death (32). Therefore, it is particularly urgent and crucial to pay attention to the nutritional status of the elderly patient population. Future research will further explore the effects of specific nutrients, dietary patterns, and nutritional interventions on cancer patients.

In addition, current tumor nutrition research is more in-depth, emphasizing systematization, personalization, and multidisciplinary cooperation. The research methods are also relatively advanced, especially in the fields of immunonutrition, metabolomics, and microbiology. Hari et al. (33) proposed that micronutrients in serum are related to tumor growth. By regulating the immune system through specific nutritional intervention means, the immune response of patients can be enhanced, and the anti-tumor treatment effect can be improved (34). In addition, metabolomics and microbiology research also provide new theoretical support for tumor nutritional intervention. The abnormal metabolism of tumor cells and the imbalance of the gut microbiota are considered to be potential mechanisms for tumor development and drug resistance (35). In the future, further exploration of how to optimize the metabolic state and gut microbiota of patients to improve their immune function and treatment tolerance will become a research hotspot.

To keep data quality manageable we restricted retrieval to WoSCC and CNKI; consequently, oncology nutrition articles indexed only in PubMed or Scopus (particularly regional journals or non-English proceedings) may have been missed, so the longitudinal trends should be read as conservative estimates. We used the CiteSpace default g-index (*k* = 25) with Pathfinder + Pruning sliced by one-year intervals to balance cluster granularity and interpretability, but different thresholds or pruning schemes could shift the relative weight of small clusters or dilute weak ties. We note this analytical bias and encourage future updates that cross-validate clusters with alternative software or parameter sweeps.

## 5 Conclusion

This study used bibliometric methods to visually analyze the literature on tumor-related nutrition research in the past 20 years, aiming to provide a reference for subsequent related research. Taken together, the bibliometric map points to three actionable gaps. First, geriatric oncology nutrition remains underpowered compared with perioperative trials in younger cohorts; upcoming calls should fund longitudinal studies that pair frailty indices with standardized nutrition screening to define trigger points for intervention. Second, immunonutrition is still treated as an optional add-on in most Enhanced Recovery pathways; policymakers and payers could pilot bundled payments that cover perioperative immune-modulating formulas, linking reimbursement to postoperative infection and length-of-stay metrics. Third, microbiome-directed nutrition (e.g., prebiotic/probiotic or fecal-microbiota-informed diets) is scarcely represented despite rising interest in immunotherapy tolerance; multicenter registries should capture diet–microbiome–outcome data to guide targeted feeding protocols. Prioritizing these areas would translate the mapped hotspots into coordinated research agendas and practical clinical pathways. In clinical practice, the nutritional status of tumor patients should be assessed and screened at an early stage. Advanced technologies and methods should be adopted to integrate nutritional support throughout the entire treatment cycle of tumor patients, helping patients complete treatment smoothly and improve their survival and quality of life.

In the future, mature experience and research results should be learned from to conduct more detailed and in-depth research. Due to the adaptability of the Cite Space software, the literature in this paper was manually screened, which has some limitations, and there may be omissions in the screening process. Secondly, only the relevant literature in the CNKI and Web of Science Core Collection databases was extracted and analyzed, so the analysis results may have certain limitations. Future research can try to combine multiple databases to more comprehensively reveal the content, hotspots, and development trends of global tumor patient nutrition nursing research.

## Data Availability

The raw data supporting the conclusions of this article will be made available by the authors, without undue reservation.
